# *Xenopus* Cdc7 executes its essential function early in S phase and is counteracted by checkpoint-regulated protein phosphatase 1

**DOI:** 10.1098/rsob.130138

**Published:** 2014-01-08

**Authors:** Wei Theng Poh, Gaganmeet Singh Chadha, Peter J. Gillespie, Philipp Kaldis, J. Julian Blow

**Affiliations:** 1 Centre for Gene Regulation and Expression, College of Life Sciences, University of Dundee, Dow St., Dundee DD1 5EH, UK; 2 Institute of Molecular and Cell Biology, Agency for Science, Technology, and Research (A*STAR), Singapore 138673, Republic of Singapore

**Keywords:** Cdc7, *Xenopus*, DNA replication, PP1, PHA-767491

## Abstract

The initiation of DNA replication requires two protein kinases: cyclin-dependent kinase (Cdk) and Cdc7. Although S phase Cdk activity has been intensively studied, relatively little is known about how Cdc7 regulates progression through S phase. We have used a Cdc7 inhibitor, PHA-767491, to dissect the role of Cdc7 in *Xenopus* egg extracts. We show that hyperphosphorylation of mini-chromosome maintenance (MCM) proteins by Cdc7 is required for the initiation, but not for the elongation, of replication forks. Unlike Cdks, we demonstrate that Cdc7 executes its essential functions by phosphorylating MCM proteins at virtually all replication origins early in S phase and is not limiting for progression through the *Xenopus* replication timing programme. We demonstrate that protein phosphatase 1 (PP1) is recruited to chromatin and rapidly reverses Cdc7-mediated MCM hyperphosphorylation. Checkpoint kinases induced by DNA damage or replication inhibition promote the association of PP1 with chromatin and increase the rate of MCM dephosphorylation, thereby counteracting the previously completed Cdc7 functions and inhibiting replication initiation. This novel mechanism for regulating Cdc7 function provides an explanation for previous contradictory results concerning the control of Cdc7 by checkpoint kinases and has implications for the use of Cdc7 inhibitors as anti-cancer agents.

## 2. Introduction

In eukaryotic cells, DNA replication occurs through a series of ordered events beginning with the binding of the origin recognition complex (ORC) to DNA. Cdc6 and Cdt1 are assembled onto ORC and promote the loading of a double hexamer of the mini-chromosome maintenance 2–7 (MCM) complex around origin DNA. This forms the pre-replicative complex (pre-RC) and licenses the origin for replication in the subsequent S phase [1–5]. The firing of licensed origins during S phase is triggered by two S phase promoting kinases, Cdk and Dbf4-dependent Cdc7 kinase (DDK), which promote assembly of the Cdc45-MCM-GINS (CMG) replicative helicase [6].

Cdc7 is an essential serine/threonine kinase conserved from yeast to humans. It is activated by associating with a cyclin-like regulatory subunit, Dbf4. In vertebrates, a second regulator of Cdc7, Drf1, has been identified [7–11]. Cdc7 phosphorylates several components of the replicative machinery, including multiple subunits of the Mcm2–7 complex [12–16], Cdc45 [17,18] and DNA polymerase α [19]. Of these proteins, Mcm2–7 appears to be the essential DDK target during DNA replication, and in budding yeast a mutation in *MCM5* can bypass the requirement for Cdc7 and Dbf4 [20]. In *Xenopus* egg extracts, Cdc7 is recruited directly to chromatin-bound Mcm2–7 by its regulatory subunit [15,21]. The N-terminus of Mcm2, Mcm4 and Mcm6 appear to be major substrates for DDK kinase activity [6]. The hyperphosphorylation of Mcm4 requires DDK activity *in vivo* and is enriched in the CMG complex. An inhibitory activity present on the Mcm4 N-terminal tail is relieved upon DDK phosphorylation [22], and DDK activity is no longer required for viability in cells lacking this inhibitory region. This suggests that the essential function of DDK is to relieve the inhibitory activity residing in the N-terminal tail of Mcm4.

It is currently unclear how DDK activity is regulated during S phase. In budding yeast, DDK is required late in S phase for the initiation of late-firing origins [23,24]. In fission yeast, Cdc7 is a rate-limiting factor for origin firing and increased levels of Cdc7 and Dbf4 enhance origin firing [25,26]. The recruitment of Cdc7 and Dbf4 to pericentromeric replication origins early in the cell cycle allows them to initiate replication early in S phase [27]. The DDK subunit Dbf4 is in low abundance in budding yeast and overexpression of Dbf4 with two CDK substrates, Sld2 and Sld3, plus their binding partner Dpb11 is sufficient to allow late-firing origins of replication to initiate early [28,29]. These studies in yeast suggest that DDK plays a role in promoting initiation at individual replication origins to drive the replication timing programme. However, studies in other organisms are preliminary, and activities that are rate-limiting for S phase progression in metazoans have not been defined.

When replication is inhibited or DNA is damaged during S phase, activation of checkpoint kinases helps to promote completion of S phase by stabilizing replication forks [30] and regulating the firing of dormant replication origins [31]. In budding yeast, phosphorylation of Dbf4 by the Rad53 checkpoint kinase plays a role in restricting origin firing [32,33]. However, the role of DDKs in the checkpoint response in metazoans is currently controversial. Initial studies suggested that the topoisomerase II (Topo II) inhibitor etoposide causes checkpoint-mediated inhibition of DDK complex formation and kinase activity [34,35]. However, later studies provided evidence that DDK expression, complex formation, chromatin association and kinase activity remain intact in cells during S phase checkpoint responses [9,11,36–38].

In this study, we have addressed aspects of DDK function in *Xenopus* egg extracts using PHA-767491 [39,40], a small molecule inhibitor of Cdc7. We show that Cdc7 phosphorylates Mcm4 and executes its essential replication function early in S phase. Unlike the case for Cdk activity, DDK activity is not limiting for progression through the replication timing programme. We demonstrate that protein phosphatase 1 (PP1) rapidly reverses DDK-mediated Mcm4 hyperphosphorylation. We also prove that checkpoint kinase activity induced by etoposide reduces Mcm4 phosphorylation but does not reduce the amount of chromatin-associated Cdc7. Finally, we show that etoposide increases the association of PP1 with chromatin in a checkpoint-dependent manner. This suggests that checkpoint-mediated recruitment of PP1 to chromatin plays a major part in the response to the inhibition of DNA replication.

## 3. Results

### 3.1. PHA-767491 inhibits DNA replication in *Xenopus* extracts

**Figure 1.**
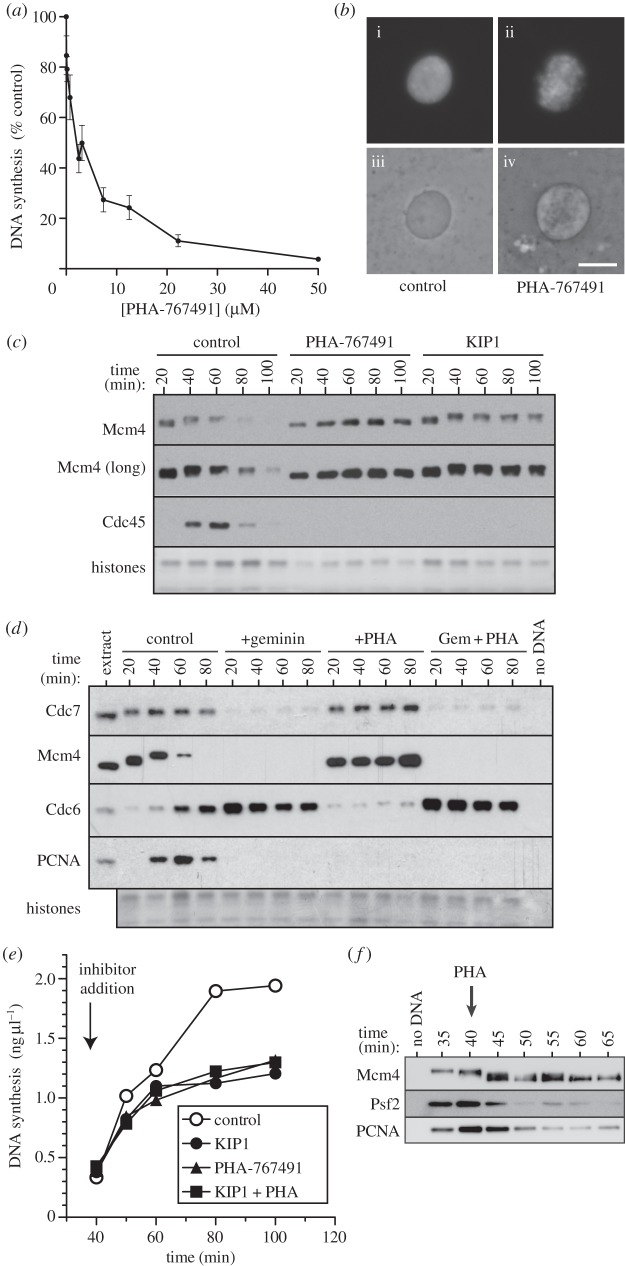
PHA-767491 inhibits DNA replication and Mcm4 hyperphosphorylation. (*a*) Interphase *Xenopus* egg extract was supplemented with demembranated sperm nuclei and [α-^32^P]dATP plus different concentrations of PHA-767491; after 90 min total DNA synthesis was determined. Mean and s.e.m. of 20 independent experiments is shown. (*b*) Extracts supplemented with demembranated sperm nuclei plus (ii,iv) or minus (i,iii) 50 μM PHA-767491 were incubated for 40 min; nuclei were then stained with Hoechst 33258 and visualized with phase contrast (iii,iv) or fluorescence microscopy (i,ii). Scale bar, 10 μm. (*c,d*) Extracts were supplemented with demembranated sperm nuclei plus or minus 50 μM PHA-767491, p27^kip1^ or geminin. After incubation for the indicated times, chromatin was isolated and immunoblotted for Mcm4, Cdc45, Cdc7, Cdc6 and PCNA. The lower portion of the gel was stained with Coomassie to visualize histones. (*e*) Egg extract was supplemented with demembranated sperm nuclei and [α-^32^P]dATP. After incubation for 40 min, aliquots were optionally supplemented with 50 μM PHA-767491, p27^kip1^ or both. At the indicated times, total DNA synthesis was determined. (*f*) Egg extract was first incubated with demembranated sperm nuclei for 40 min, and the extract was supplemented with 50 μM PHA-767491. At the indicated times, chromatin was isolated and immunoblotted for Mcm4, Psf2 and PCNA.

We titrated PHA-767491 [39,40] into *Xenopus* egg extracts and measured its effect on the replication of demembranated *Xenopus* sperm nuclei. About 20–50 µM PHA-767491 fully inhibited DNA synthesis (figure 1*a*). Nuclear envelope formation still took place in the presence of PHA-767491, indicating that the inhibition of DNA synthesis was not due to non-specific effects (figure 1*b*). Similar results were obtained using G1 nuclei from somatic Chinese hamster ovary (CHO) cells as DNA template (see electronic supplementary material, figure S1*a*).

Cdc7-mediated phosphorylation can be observed as a mobility shift of Mcm4 after SDS–PAGE [9,22,41] (see electronic supplementary material, figure S1*b*). Hyperphosphorylation of chromatin-bound Mcm4 was inhibited when extracts were treated with 50 µM PHA-767491 but was not affected when Cdk activity was blocked with 100 nM p27^kip1^ (figure 1*c* and the electronic supplementary material, figure S1*c*). This is consistent with previous reports showing that the essential replication function of Cdc7 is executed before that of Cdks [15,42,43]. PHA-767491 inhibited the association of Cdc45 and proliferating cell nuclear antigen (PCNA) with chromatin, indicating that the initiation of replication forks was inhibited (figure 1*c,d* and the electronic supplementary material, figure S1*c*). In previous work, 1.4 µM PHA-767491 was reported to inhibit recombinant Cdc7 kinase by 90% [44]. Consistent with this value, we observed that 3 µM PHA-767491 was required to substantially inhibit Mcm4 hyperphosphorylation in *Xenopus* extract and this correlated with the reduction of chromatin-bound Cdc45 and PCNA (see electronic supplementary material, figure S1*d*). Because of the complex nonlinear relationship between the rate of replication initiation and total DNA synthesis, higher concentrations of PHA-767491 are required to abolish DNA replication in *Xenopus* egg extract.

Cdc7 is recruited to chromatin by direct interaction with the Mcm2–7 double hexamer [15,21], which does not occur when licensing is prevented by treating extracts with geminin (figure 1*d*). In contrast, no significant decrease in Cdc7 chromatin association was seen in extracts treated with PHA-767491 (figure 1*d*). In addition, PHA-767491 did not block the association of Cdc6 with chromatin that occurs when licensing is blocked [45–47] (figure 1*d*). Taken together, these results are consistent with the idea that PHA-767491 inhibits the initiation of DNA replication by blocking the kinase activity of Cdc7.

Previous work has demonstrated that in *Xenopus* egg extracts, S phase Cdks (Cdk2/cyclin E and Cdk2/cyclin A) are required throughout S phase for the initiation of replication but are not required for fork progression [48–50]. When CDK inhibitors such as p27^kip1^ are added to extracts in early S phase the rate of DNA synthesis drops off over a period of 15–20 min as forks terminate within active origin clusters [49] (figure 1*e*, filled circles). When PHA-767491 was added to extract in early S phase, it had a similar effect to p27^kip1^, suggesting that, like Cdks, Cdc7 is required for ongoing initiation throughout S phase (figure 1*e*, squares). This is consistent with DNA fibre analysis in human cells suggesting that PHA-767491 does not significantly inhibit replication fork progression [39].

When we examined the chromatin in these experiments, we observed that Mcm4 was almost completely dephosphorylated within 10 min after PHA-767491 addition to the extract (figure 1*f*). This raises the possibility that Cdc7-dependent MCM phosphorylation is required for both initiation and elongation of replication forks, and that the decline in replication rate seen when PHA-767491 is added to extracts in early S phase is partly due to decreasing fork progression rates as MCM dephosphorylation occurs. However, two features of this experiment argue against a role for Cdc7-dependent MCM phosphorylation in fork elongation. First, if Cdc7 activity were required for elongation of existing forks, then co-addition of PHA-767491 with p27^kip1^ in early S phase should cause more inhibition of DNA synthesis over that seen with p27^kip1^ alone; however, this was not the case (figure 1*e*, triangles). Second, the decline in replication rate seen after addition of PHA-767491 was associated with a corresponding loss of chromatin-bound Psf2 (part of the GINS complex) and PCNA (a processivity factor for replicative polymerases), consistent with the decline in replication rate being due to termination of active replisomes (figure 1*f*).

**Figure 2.**
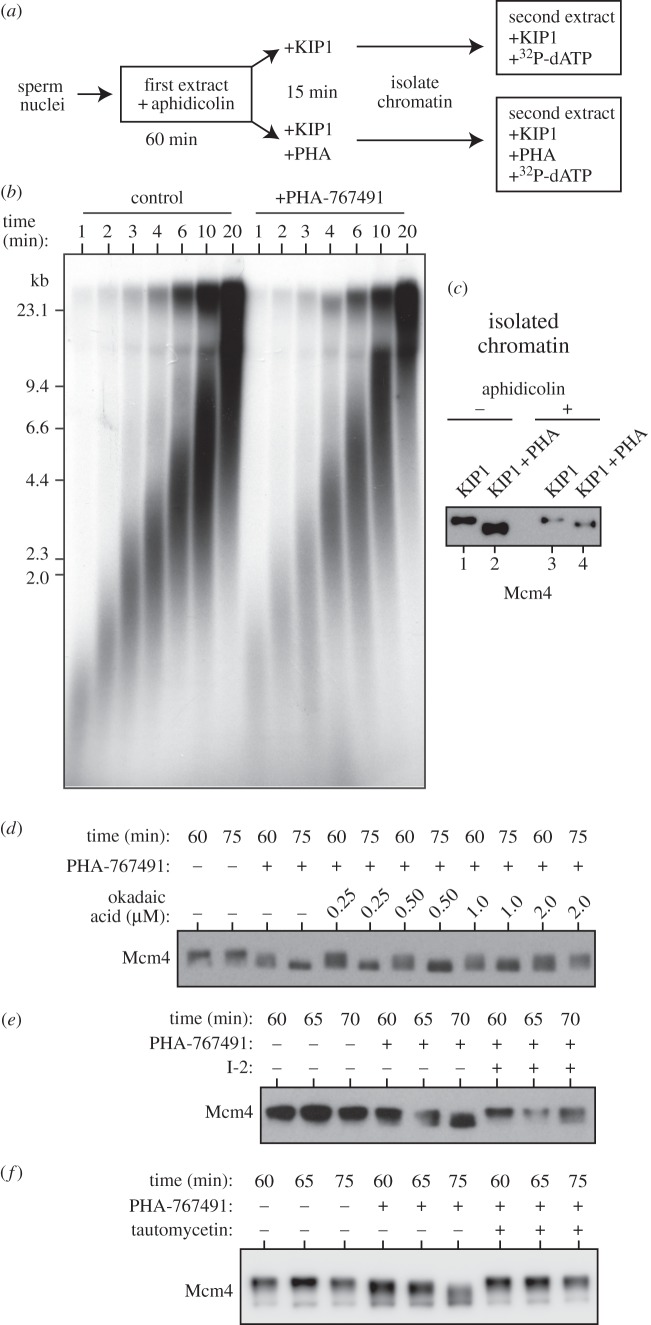
Fork rate and the reversal of Mcm4 hyperphosphorylation by PP1. (*a–c*) Sperm nuclei were incubated in egg extract supplemented with 100 µM aphidicolin. After 60 min incubation, one aliquot was supplemented with p27^kip1^ and one aliquot was supplemented with p27^kip1^ plus 50 μM PHA-767491. After a further 15 min incubation chromatin was isolated. Chromatin was then incubated in extract supplemented with [α-^32^P]dATP and p27^kip1^ and optionally with 50 μM PHA-767491 to match the first incubation. (*a*) Cartoon of experimental set-up. (*b*) At the indicated times, DNA was isolated, separated on an alkali agarose gel and autoradiographed. Molecular weight markers (in kb) are shown to the left. (*c*) Chromatin isolated after the first incubation was immunoblotted for Mcm4 (lanes 3 and 4). Chromatin from a parallel incubation lacking aphidicolin was loaded as a comparison (lanes 1 and 2). (*d–f*) Sperm nuclei were incubated for 60 min in extracts treated with p27^kip1^ to allow Mcm4 hyperphosphorylation. PHA-767491 (50 μM) was then added, and chromatin was isolated either immediately after PHA-767491 addition (60 min) or 5 or 15 min later (65 or 75 min, respectively). Extract was optionally supplemented with the indicated concentrations of okadaic acid at the time of PHA-767491 addition (*d*) 1.2 μM I-2 45 min after sperm addition (*e*) or 1 µM tautomycetin at the time of PHA-767491 addition (*f*).

We directly addressed whether Cdc7-dependent MCM phosphorylation is required for fork elongation with the experiment outlined in figure 2*a*. Sperm nuclei were incubated in extract in the presence of 100 µM aphidicolin, a competitive inhibitor of replicative DNA polymerases, which allows forks to initiate but prevents them from moving away from replication origins. After 60 min, extracts were supplemented with p27^kip1^ to prevent any further initiation, and optionally supplemented with PHA-767491. After a further 15 min incubation, Mcm4 remained fully phosphorylated in the presence of p27^kip1^ alone, but had become dephosphorylated in extracts supplemented with PHA-767491 (figure 2*c*). Chromatin was then isolated and transferred to extract containing p27^kip1^ (to prevent any further initiation events from occurring), and optionally supplemented with PHA-767491 to prevent re-phosphorylation of the dephosphorylated MCMs. Nascent DNA was labelled with [α-^32^P]dATP and then separated on alkali agarose gels to monitor replication fork progression (figure 2*b*). Forks containing phosphorylated and unphosphorylated Mcm4 progressed at essentially the same rate: 658 ± 54 nt min^−1^ for the control sample and 698 ± 33 nt min^−1^ for the PHA-767491 sample (mean ± s.e.m. for three independent experiments). This suggests that MCM phosphorylation by Cdc7 is not required for efficient fork elongation once initiation has occurred. Some smearing of the nascent strands in the PHA-767491 samples may suggest a slight decrease of fork stability in the absence of Cdc7 activity.

### 3.2. PP1 reverses Mcm4 hyperphosphorylation

Figures 1*f* and 2*c* demonstrate that Mcm4 is rapidly dephosphorylated after Cdc7 activity is inhibited, therefore we investigated how this happens. Sperm chromatin was incubated in *Xenopus* extract supplemented with p27^kip1^ to allow hyperphosphorylated Mcm4 to accumulate on chromatin. When PHA-767491 was then added, Mcm4 was rapidly dephosphorylated (figure 2*d*). Okadaic acid is an inhibitor of protein phosphatases in the PP1 and PP2A classes with a higher potency towards PP2A, so that submicromolar concentrations typically inhibit PP2A but not PP1. When high concentrations (2 µM) of okadaic acid were added along with PHA-767491, Mcm4 dephosphorylation was abolished; however, lower concentrations (0.25–1 µM) of okadaic acid were largely ineffective (figure 2*d*). This suggests that PP1 is largely responsible for the rapid dephosphorylation of Mcm4. Consistent with this idea, PP1 has been shown to physically interact with Mcm2–7 hexamers bound to chromatin in *Xenopus* egg extracts [5]. To test the role of PP1 directly, we optionally treated extracts with Inhibitor-2 (I-2), a peptide inhibitor of PP1 (figure 2*e*), or tautomycetin, another PP1-selective inhibitor (figure 2*f*). Both of these inhibitors prevented the dephosphorylation of Mcm4, suggesting that PP1 is the main phosphatase acting on Mcm4 in *Xenopus* extracts.

**Figure 3.**
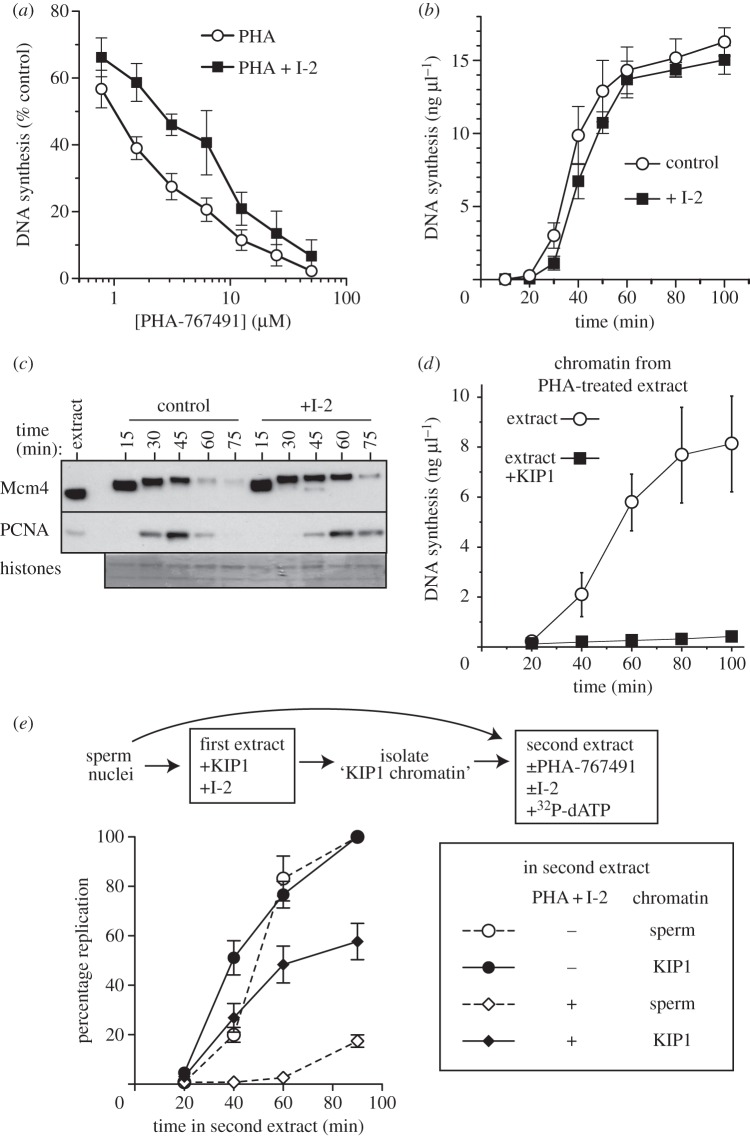
Effects of I-2, PHA-767491 and Cdk activity on DNA replication. (*a*) Interphase *Xenopus* egg extract was supplemented with demembranated sperm nuclei, [α-^32^P]dATP and different concentrations of PHA-767491, plus or minus I-2; after 90 min total DNA synthesis was determined. Mean and s.e.m. of three independent experiments is shown. (*b*) Extract was supplemented with sperm nuclei at 15 ng DNA μl^−1^, [α-^32^P]dATP and optionally with I-2. At the indicated times, total DNA synthesis was measured. Mean and s.e.m. of three independent experiments is shown. (*c*) Extract was supplemented with sperm nuclei at 10 ng DNA μl^−1^ and optionally with I-2. At the indicated times, chromatin was isolated and immunoblotted for Mcm4 and PCNA. The lower portion of the gel was stained with Coomassie to visualize histones. (*d*) Chromatin was prepared by incubating sperm nuclei at 20 ng DNA μl^−1^ in interphase extract supplemented with 50 μM PHA-767491; after 60 min chromatin was isolated and transferred to fresh extract containing [α-^32^P]dATP optionally supplemented with p27^kip1^. At different times, aliquots were taken, and total DNA synthesis was determined. Mean and s.e.m. of three independent experiments is shown. (*e*) ‘KIP1 chromatin’ was prepared by incubating sperm nuclei at 20 ng DNA μl^−1^ in interphase extract supplemented with p27^kip1^ and 1.2 μM I-2; after 40 min, chromatin was isolated. ‘KIP1 chromatin’ or naive sperm nuclei were then incubated in fresh extract containing [α-^32^P]dATP, optionally supplemented with 20 μM PHA-767491 and 1.2 μM I-2. At different times, aliquots were taken, and total DNA synthesis was determined and expressed as percentage of the amount synthesized by 90 min in control (untreated extract). Mean and s.e.m. of three independent experiments is shown.

In order to demonstrate a functional role for PP1 in dephosphorylating MCMs, we titrated PHA-767491 into egg extract plus or minus I-2. Inhibition of PP1 made DNA synthesis more resistant to PHA-767491 (figure 3*a*), consistent with PP1 playing a significant role in reversing Cdc7 action. Despite this, the balance between PP1 and Cdc7 strongly favours the phosphorylated state as Mcm4 hyperphosphorylation is seen soon after Cdc7 is recruited to chromatin (figure 1*d*). Consistent with this conclusion, inhibition of PP1 activity by pre-incubating extract with I-2 did not cause observable acceleration of DNA synthesis (figure 3*b*) or Mcm4 hyperphosphorylation (figure 3*c*). Instead, we observed that higher concentrations of I-2 delayed or inhibited DNA synthesis, suggesting that PP1 displays additional positive roles in DNA replication (see electronic supplementary material, figure S1*e*). This is consistent with other reported functions of PP1 in *Xenopus* extracts, which include the reversal of ATM activity [51,52].

### 3.3. Specificity of PHA-767491

We next determined whether the inhibition of DNA replication by PHA-767491 was solely due to inhibition of Cdc7 activity. When tested against a panel of 95 protein kinases, 1 µM PHA-767491 inhibited only seven other protein kinases greater than 90%: DYRK1A, 2 and 3, PRK2, GSK3β, p38δ MAPK and CK1 (see electronic supplementary material, table S1), broadly in line with previous reports [39,40,44]. None of these kinases have been implicated in the initiation of DNA replication, and inhibitors of these kinases did not significantly inhibit DNA replication in egg extracts (data not shown). The only kinase previously implicated in DNA replication that is affected by PHA-767491 is Cdk2, which was inhibited by 72% under these assay conditions.

We designed experiments to test the extent to which PHA-767491 inhibits DNA replication by inhibiting Cdk2 activity, exploiting the observation that Cdc7 acts before Cdks to promote DNA replication [15,42]. We first confirmed that PHA-767491 inhibits initiation prior to the last Cdk-dependent step. Sperm nuclei were incubated in extract containing PHA-767491 and then chromatin was transferred to extract plus or minus p27^kip1^ to inhibit Cdks (figure 3*d*). Replication occurred only in the absence of p27^kip1^, demonstrating that the Cdk-dependent step in replication initiation cannot occur prior to the step inhibited by PHA-767491.

Sperm nuclei were then incubated in extracts containing p27^kip1^, which allows Mcm4 to be hyperphosphorylated while preventing replication initiation by inhibiting S phase Cdks. I-2 was added to minimize dephosphorylation of Mcm4. ‘KIP1 chromatin’ was then isolated and transferred to fresh extract containing [α-^32^P]-dATP and optionally containing 20 µM PHA-767491 and I-2 (figure 3*e*). Replication then depends on Cdk activity in the second extract, so if S phase Cdk activity were inhibited by 20 µM PHA-767491, no DNA replication will take place. Figure 3*e* shows that pre-incubated ‘KIP1 chromatin’ replicated to approximately 60% of control levels in extract treated with PHA-767491. This suggests that the Cdk-dependent step in DNA replication can occur in the presence of concentrations of PHA-767491 that inhibit replication initiation. The failure of pre-incubated ‘KIP1 chromatin’ to completely replicate in extract containing PHA-767491 is most likely due to some MCM dephosphorylation occurring even in the presence of I-2; higher I-2 concentrations could not be used because they inhibit DNA replication (see electronic supplementary material, figure S1*e*).

### 3.4. Time of action of Cdc7

We next used PHA-767491 to investigate the execution point of Cdc7 in S phase. We first established that unlike Mcm4 that is bound to DNA, soluble Mcm4 was not hyperphosphorylated and its mobility on SDS–PAGE was not significantly changed when extracts were treated with PHA-767491 (see electronic supplementary material, figure S2*a*). Once origins have been licensed by loading Mcm2–7, chromatin is then assembled into interphase nuclei [53]. Although Cdc7 can be recruited to licensed chromatin prior to nuclear assembly [15], this occurs more efficiently after nuclear assembly has taken place [42]. Using wheat germ agglutinin (WGA) to disrupt nuclear pore function [54], we demonstrated that increasing concentrations of WGA progressively inhibited both Mcm4 hyperphosphorylation and Cdc7 chromatin association (see electronic supplementary material, figure S2*b*) at concentrations that inhibited both DNA synthesis (see electronic supplementary material, figure S2*c*) and nuclear assembly (see electronic supplementary material, figure S2*d*). This indicates that the nuclear import of Cdc7 enhances, though is not essential for, Cdc7 binding and phosphorylation of chromatin-bound Mcm2–7.

When sperm nuclei replicate in *Xenopus* egg extracts, origins initiate over a period of 20–30 min [49,55,56]. In order to determine whether Cdc7 acts on all origins early in S phase, or whether it acts on late-firing origins only later in S phase, we used a hybrid system where mammalian somatic nuclei from cells synchronized in G1 are incubated in *Xenopus* extracts [57,58]. These nuclei replicate in *Xenopus* extract according to the same timing programme observed *in vivo*, though compressed into a shorter interval of approximately 120 min compared with the approximately 8 h measured *in vivo*. This provides a longer S phase compared with sperm nuclei, making it easier to distinguish different stages of S phase.

**Figure 4.**
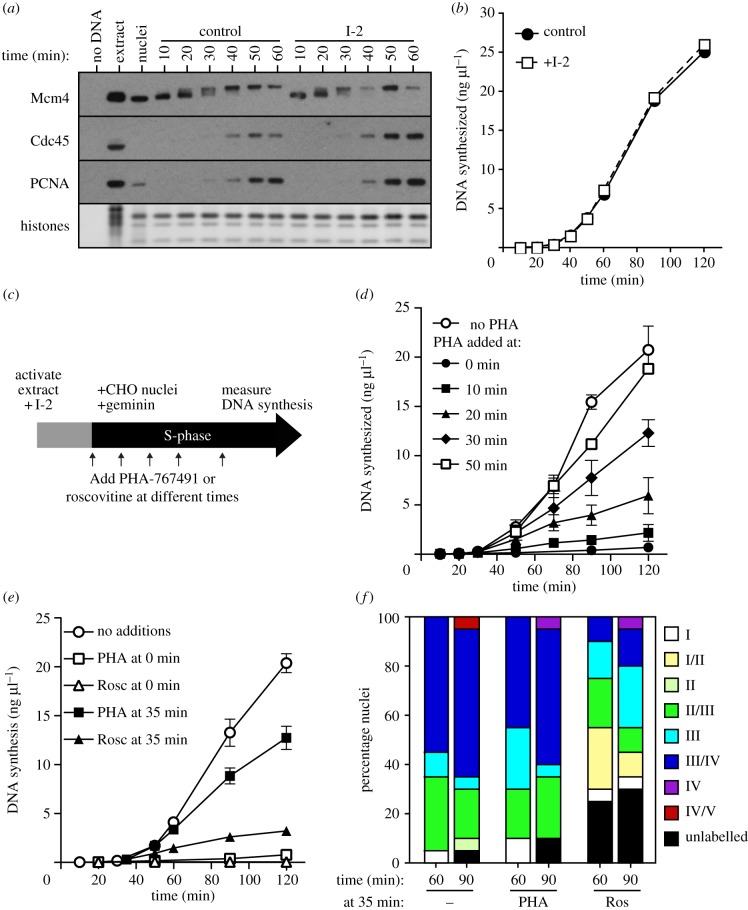
Cdc7 fulfills its essential function early in S phase. (*a,b*) G1 CHO nuclei were incubated in interphase extract supplemented with geminin and optionally supplemented with I-2 or [α-^32^P]dATP. At the indicated times, chromatin was isolated and immunoblotted for Mcm4 (*a*) or DNA synthesis was determined by measuring [α-^32^P]dATP incorporation (*b*). (*c–f*) Interphase egg extracts were supplemented with G1 CHO nuclei, geminin and I-2 and optionally with [α-^32^P]dATP. At different times thereafter extract was optionally supplemented with 20 μM PHA-767491 or 1 mM roscovitine. (*c*) Schematic of experiments. (*d,e*) At the indicated times, total DNA synthesis was determined. (*f*) At 60 or 90 min, extract was pulsed with Cy3-dUTP and sites of DNA synthesis were visualized by fluorescence microscopy. The proportion of nuclei showing different replication patterns (I–V; representing stages from early through late S phase) was determined. The mean of three independent experiments (*n* = 20 nuclei for each sample) is plotted [58]. Type I, faintly punctate labelling throughout euchromatic regions; type II, complete diffuse labelling of euchromatic regions, with lack of nucleolar labelling; type III, intense labelling of the peripheral ring; type IV, labelling of small speckled heterochromatic foci within the nuclear interior or at the periphery; type V, predominant labelling of large internal replication foci and at the periphery of the nucleus.

When nuclei from G1 CHO cells were added to *Xenopus* extract, chromatin-bound Mcm4 became maximally hyperphosphorylated within 20–40 min (figure 4*a*), slightly slower than was observed using *Xenopus* sperm nuclei (see electronic supplementary material, figure S1*b*). However, on both templates, maximal Mcm4 hyperphosphorylation occurred in early S phase, suggesting that by this time Cdc7 has acted on both early and late origins. Addition of the PP1 inhibitor I-2 did not accelerate either Mcm4 hyperphosphorylation (figure 4*a*) or the rate of DNA synthesis (figure 4*b*), consistent with our observations on sperm nuclei (figure 3*b,c*). To show that Cdc7 executes its essential function at all replication origins early in S phase, we added PHA-767491 to extracts at different times after addition of CHO nuclei (figure 4*c,d*). I-2 was present in all experiments to prevent dephosphorylation of Mcm2–7. Figure 4*d* shows that when PHA-767491 was added at 30 min, before S phase had started, replication subsequently occurred to approximately 60% of control levels. When PHA-767491 was added at 50 min, in very early S phase, subsequent DNA synthesis occurred to approximately 90% of control levels. This suggests that the essential function of Cdc7 at both early- and late-firing origins is completed very early in S phase and correlates with the time of Mcm4 hyperphosphorylation, even though dephosphorylation of Mcm4 by PP1 can occur subsequently.

These results are in contrast to experiments indicating that even late in S phase, inhibition of Cdk activity prevents further initiation [49,58]. To verify this difference between Cdk and Cdc7 execution points, we compared PHA-767491 with a Cdk inhibitor, roscovitine, in extracts where MCM dephosphorylation was prevented by the addition of I-2. When PHA-767491 or roscovitine was added at the same time as the CHO nuclei, DNA replication was completely inhibited (figure 4*e*, open symbols). When PHA-767491 was added at 35 min, replication proceeded to 63% of control levels (figure 4*e*, filled squares), consistent with the results shown in figure 4*d*. By contrast, when roscovitine was added at 35 min, replication was largely inhibited (to 16% of control levels; figure 4*e*, filled triangles) because Cdks only act at origins less than 5 min before they initiate [49]. The more pronounced effect of roscovitine on the replication of CHO nuclei when compared with sperm chromatin (compare figures 1*e* and 4*e*) is because of the more extended timing programme of the CHO nuclei [58] meaning that initiation events occur over a much longer period of time. These results indicate that in *Xenopus* egg extracts Cdc7 executes its function before Cdk, and suggest that once Cdc7 has completed its essential function it is no longer the rate-limiting factor in driving the replication timing programme. However, continued Cdc7 activity may still be required later in S phase because of the PP1-mediated dephosphorylation of Mcm4. Differences between the activity of roscovitine and PHA-767491 in this experiment also provide further evidence that, at the concentration used, PHA-767491 does not substantially inhibit S phase Cdk function.

In order to study the role of Cdc7 in the replication timing programme progression, we analysed the different replication patterns observed as CHO nuclei progress through S phase *in vitro* [57,58]. G1 CHO nuclei were incubated in extract where PP1 had been inhibited with I-2 and then PHA-767491 or roscovitine was added at 35 min. Extracts were pulsed with Cy-3 dUTP at 60 or 90 min to allow the visualization of actively replicating factories/clusters [58]. In the absence of PHA-767491 or roscovitine, most nuclei isolated at 60 min showed a type III/IV replication pattern typical of mid–late S phase (figure 4*f*). When roscovitine was added at 35 min, the replication profile was significantly slowed relative to control (*p* < 0.0001 by Mann–Whitney test at both 60 and 90 min), indicating that continued Cdk activity during S phase is required for progression through the replication timing programme [58]. In contrast, when PHA-767491 was added at 35 min, the overall replication pattern profile was not significantly different from the controls (*p* ≥ 0.1 by Mann–Whitney test at both 60 and 90 min), indicating that continued Cdc7 activity is not required for progression of nuclei through the replication timing programme if Mcm2–7 dephosphorylation is prevented.

### 3.5. Effect of etoposide on Cdc7 function

**Figure 5.**
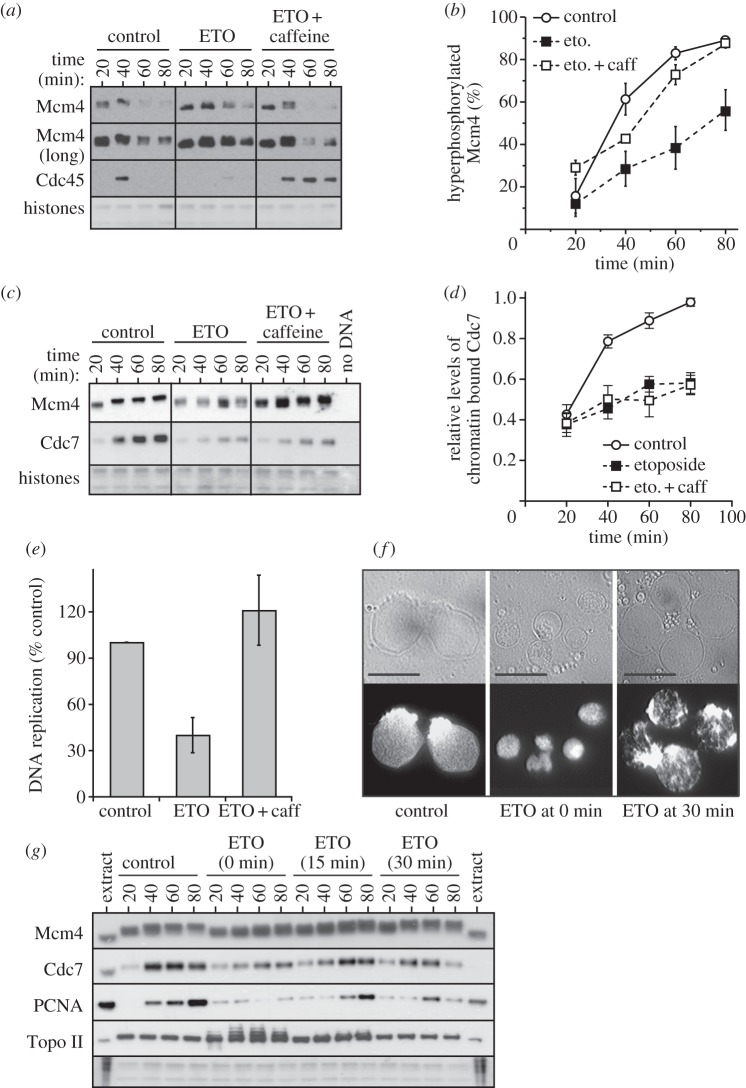
Early addition of etoposide reduces Cdc7 activity on chromatin. (*a–e*) Sperm nuclei were incubated at 10 ng DNA μl^−1^ in extracts treated with 300 μM etoposide (ETO) and/or 5 mM caffeine. (*a,c*) At the indicated times, chromatin was isolated and immunoblotted for Mcm4, Cdc7 and Cdc45. The lower portion of the gel was stained with Coomassie to visualize histones. (*b*) The percentage of Mcm4 hyperphosphorylation was quantified in three independent experiments. The mean and s.e.m. is shown. (*d*) Cdc7 levels were quantified and expressed as a proportion of the peak value. Mean and s.e.m. for six independent experiments are shown. (*e*) Extracts were also supplemented with [α-^32^P]dATP and after 90 min total DNA synthesis was determined. (*f,g*) 10 ng DNA μl^−1^ sperm nuclei were incubated in extracts, and at 0, 15 or 30 min after DNA addition extract was optionally supplemented with 300 μM etoposide. (*f*) After 90 min, nuclei were stained with Hoechst 33258 and visualized by phase contrast (top) or fluorescence microscopy (bottom). Scale bar, 25 μm. (*g*) At the indicated times, chromatin was isolated and immunoblotted for Mcm4, Cdc7, PCNA and Topo II.

There is some controversy over the potential regulation of Cdc7 by checkpoint kinases. A previous study has implicated Cdc7 in the intra-S checkpoint response to etoposide in *Xenopus* extracts [34]. However, subsequent work indicated that etoposide does not inhibit Cdc7 kinase activity in either *Xenopus* egg extracts or in human cells [9,11,36–38]. Consistent with the report of Costanzo *et al*. [34], we observed that when 300 µM etoposide was added to *Xenopus* extract along with sperm chromatin, Mcm4 hyperphosphorylation was inhibited (figure 5*a–c*), the amount of Cdc7 associated with chromatin decreased (figure 5*c,d*) and DNA replication was inhibited (figure 5*e* and the electronic supplementary material, figure S3*a*). When caffeine was added to abolish ATM and ATR checkpoint kinase activity, Mcm4 hyperphosphorylation (figure 5*a–c*) and DNA synthesis (figure 5*e*) were restored to control levels in etoposide-treated extract. However, consistent with Costanzo *et al*. [34], caffeine did not restore normal levels of Cdc7 on chromatin (figure 5*c,d*). This suggests that etoposide has two distinct effects on Cdc7 activity: a checkpoint-dependent inhibition of Mcm4 phosphorylation and a checkpoint-independent inhibition of Cdc7 chromatin association. Inhibition of checkpoint kinases can restore Mcm4 hyperphosphorylation even though levels of chromatin-associated Cdc7 remain low, indicating that the etoposide-induced dephosphorylation of Mcm4 is at least in part mediated by caffeine-sensitive checkpoint kinases.

To understand the checkpoint-independent effect of etoposide, we investigated its known target, Topo II, and its associated functions. Etoposide causes the accumulation of covalently linked protein–DNA complexes between Topo II and DNA. Topo II is rapidly loaded onto sperm chromatin as it undergoes decondensation in *Xenopus* egg extract [59]. Nuclei assembled in the presence of etoposide (figure 5*f*, ‘ETO at 0 min’) were significantly smaller than control nuclei. However, when etoposide was added after chromatin had decondensed, nuclei subsequently attained normal sizes (figure 5*f*, ‘ETO at 30 min’) and trapped less Topo II on DNA, allowed more Cdc7 to bind to chromatin and allowed normal levels of Mcm4 hyperphosphorylation (figure 5*g*). This is consistent with previous results indicating that proper nuclear assembly enhances Cdc7 chromatin recruitment [42] (electronic supplementary material, figure S2).

**Figure 6.**
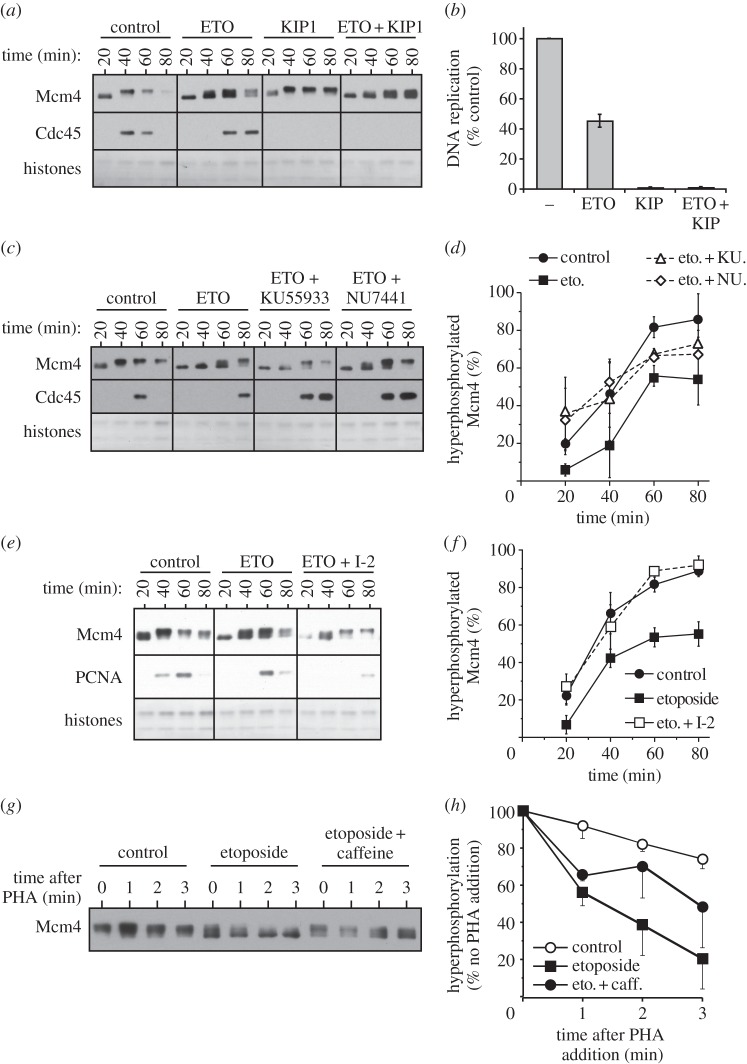
The checkpoint response to etoposide promotes Mcm4 dephosphorylation. (*a–f*) Sperm nuclei were incubated in extracts optionally treated with 300 μM etoposide, 5 mM caffeine, 100 nM p27^kip1^, 10 μM KU55933, 10 μM NU7441 and/or 1.2 μM I-2. (*a,c,e*) At the indicated times, chromatin was isolated and immunoblotted for Mcm4, Cdc45 or PCNA. The lower portion of the gel was stained with Coomassie to visualize histones. (*b*) Extract was also supplemented with [α-^32^P]dATP. After incubation for 90 min, the total amount of DNA synthesized was determined by scintillation counting. (*d,f*) The percentage of Mcm4 hyperphosphorylation was quantified in three independent experiments. The mean and s.e.m. is shown. (*g,h*) Sperm nuclei were incubated in extract optionally supplemented with etoposide and/or caffeine for 60 min. Extract was then supplemented with 50 μM PHA-767491, and at the indicated times thereafter chromatin was isolated and immunoblotted for Mcm4. (*h*) The proportion of Mcm4 that was hyperphosphorylated was quantified. Mean and s.e.m. of three independent experiments is shown. The degree of dephosphorylation induced by etoposide varied between the three experiments, but within each experiment the rate of dephosphorylation was reduced by co-addition of caffeine.

Etoposide can potentially induce extensive double-strand breaks when replication forks encounter covalently linked Topo II on DNA. We therefore examined whether etoposide requires ongoing DNA replication to inhibit Mcm4 hyperphosphorylation. Figure 6*a,b* shows that etoposide still inhibited Mcm4 hyperphosphorylation in extract supplemented with p27^kip1^ to block the initiation of DNA replication. Furthermore, creation of DNA double-strand breaks by adding a restriction enzyme to egg extract did not inhibit Mcm4 hyperphosphorylation (see electronic supplementary material, figure S3*b,c*). Taken together, these results suggest that the major effect of etoposide on Cdc7 does not require ongoing DNA synthesis.

We then asked how caffeine could rescue the etoposide-induced inhibition of Mcm4 phosphorylation without restoring normal levels of chromatin-bound Cdc7. Because caffeine is a relatively non-specific inhibitor of ATM/ATR family kinases, we used more specific inhibitors, KU55993 and NU7441, to bypass the effect of the ATM and DNA-PK kinases, respectively. Figure 6*c,d* shows that KU55993 and NU7441 were each able to partially restore Mcm4 hyperphosphorylation. The total amount of DNA synthesized was also partially rescued by KU55993 and NU7441, but recruitment of Cdc7 was not affected (see electronic supplementary material, figure S3*d,e*). This suggests that both ATM and DNA-PK play a role in etoposide-induced inhibition of Mcm4 hyperphosphorylation.

If checkpoint kinases do not inhibit Cdc7 chromatin loading (figure 5*c,d*), then they could instead promote Mcm4 dephosphorylation. Figure 6*e,f* shows that pre-treatment of extracts with the PP1 inhibitor I-2 completely prevented the etoposide-induced inhibition of Mcm4 hyperphosphorylation. Etoposide-induced inhibition of Mcm4 hyperphosphorylation is therefore sensitive to PP1 activity levels. Interestingly, I-2 treatment did not rescue the etoposide-induced inhibition of DNA replication (see electronic supplementary material, figure S3*f*), consistent with previous observations that ATM can reduce DNA replication initiation in *Xenopus* egg extracts by inhibiting Cdk2 [60].

It was previously reported that etoposide treatment of *Xenopus* egg extracts does not decrease Cdc7 kinase activity [38], consistent with the idea that checkpoint kinases inhibit Cdc7 function by increasing the rate of MCM dephosphorylation. We tested this idea by incubating chromatin in extracts optionally supplemented with etoposide or etoposide plus caffeine. At 60 min, PHA-767491 was added to the extracts, and chromatin was isolated at different times thereafter to assess the rate of Mcm4 dephosphorylation (figure 6*g,h*). On addition of PHA-767491 to control extract, Mcm4 displayed a modest decrease in phosphorylation over a period of 3 min. In etoposide-treated extract, however, Mcm4 was almost completely dephosphorylated within the same period of time. This etoposide-induced dephosphorylation was partially blocked by co-addition of caffeine. These results are consistent with the idea that etoposide reduces Mcm4 phosphorylation at least in part by inducing a checkpoint-dependent increase in the rate of Mcm4 dephosphorylation.

### 3.6. Checkpoint-dependent recruitment of PP1 to chromatin

**Figure 7.**
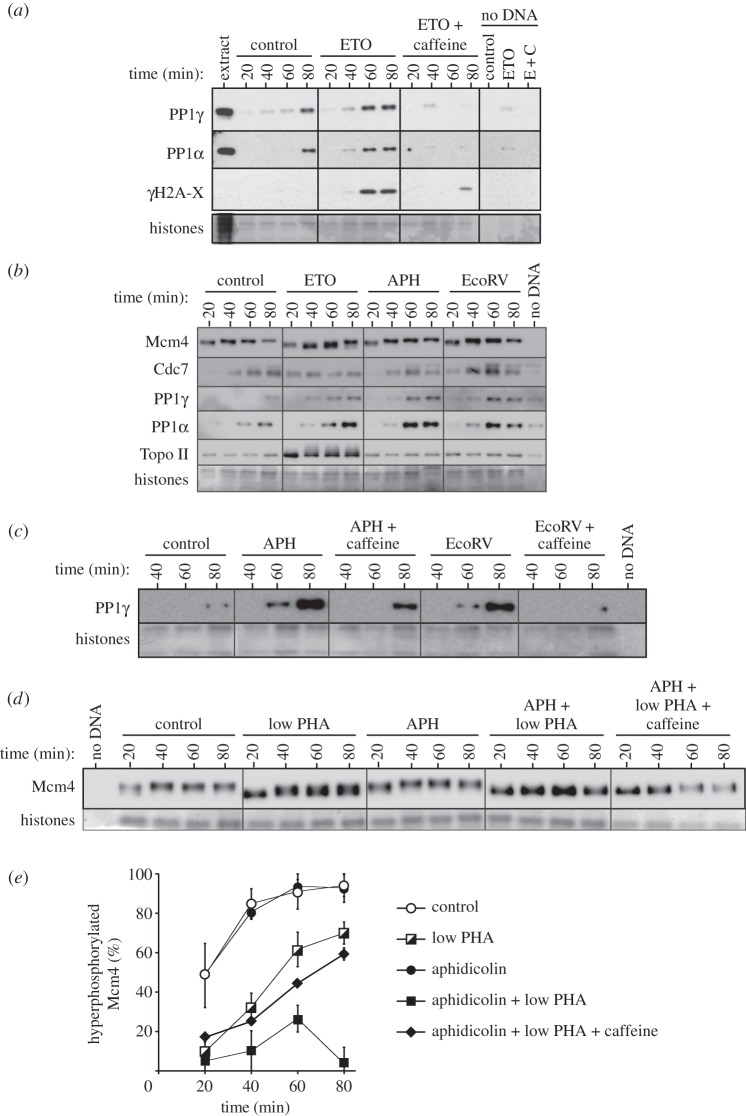
Checkpoint-dependent Mcm4 dephosphorylation and PP1. (*a–c*) Sperm nuclei were incubated in extracts optionally treated with etoposide, aphidicolin, EcoRV and 5 mM caffeine. At the indicated times, chromatin was isolated and immunoblotted for Mcm4, PP1α, PP1γ and γH2A-X. The bottom part of each gel was stained with Coomassie to visualize histones. (*d,e*) Sperm nuclei were incubated in extracts optionally treated with different combinations of 0.75 µM PHA-767491, 100 µM aphidicolin and 5 mM caffeine. At the indicated times, chromatin was isolated and immunoblotted for Mcm4. The bottom part of each gel was stained with Coomassie to visualize histones. The percentage of Mcm4 hyperphosphorylation was quantified in three independent experiments; the mean and s.e.m. is shown (*e*).

We have previously demonstrated that PP1 is the major Mcm4 phosphatase that opposes Cdc7 function (figure 2*b–d*). We therefore tested the idea that the checkpoint-dependent increase in Mcm4 dephosphorylation was mediated by increased recruitment of PP1 to chromatin. Figure 7*a* shows that etoposide strongly enhances the chromatin recruitment of two PP1 isoforms, PP1α and PP1γ. The recruitment of PP1α and PP1γ was abolished when checkpoint kinases were inhibited by caffeine, indicating that checkpoint kinases promote PP1 association with chromatin. Taken together, these results suggest that checkpoint kinase activity induced by etoposide reduces Cdc7 function by enhancing the activity of PP1 against chromatin-bound Mcm2–7.

We next examined whether the recruitment of PP1γ to chromatin was enhanced by other agents that activate checkpoint kinases. Chromatin was isolated from extract supplemented with aphidicolin, an inhibitor of replicative DNA polymerases which typically activates ATR, or from extract supplemented with the restriction enzyme EcoRV, which creates double-strand DNA breaks and activates ATM. Figure 7*b* demonstrates that both these agents enhanced the recruitment of PP1γ to chromatin to a level at least as high as is seen with etoposide. Figure 7*c* shows that the enhanced recruitment of PP1γ to chromatin induced by aphidicolin and EcoRV was largely abolished by co-addition of caffeine, suggesting that it is dependent on the activity of checkpoint kinases.

Despite the enhanced recruitment of PP1γ induced by aphidicolin and EcoRV, there was no observable decrease in Mcm4 hyperphosphorylation (figure 7*b*) comparable with that seen with etoposide. We therefore wanted to know what is unique about etoposide. We considered the possibility that etoposide is able to induce observable Mcm4 dephosphorylation, because unlike the other treatments that activate ATM and ATR, it also causes a reduction in the quantity of chromatin-bound Cdc7 in an AMT/ATR-independent manner (figure 5*b,c*). Because Cdc7 activity appears to be in large excess in the *Xenopus* system (as evidenced by the rapid and quantitative hyperphosphorylation of Mcm4 very early in S phase), significant Mcm4 dephosphorylation may require both the checkpoint-dependent increase in Mcm4 dephosphorylation and the checkpoint-independent reduction in chromatin-associated Cdc7 kinase activity. To test this idea, we partially inhibited Cdc7 activity with a very low concentration of PHA-767491 (0.75 µM) which only induced partial Mcm4 dephosphorylation (figure 7*d,e*, ‘low PHA’). A combination of 0.75 µM PHA-767491 with aphidicolin caused almost complete dephosphorylation of Mcm4 (figure 7*d,e*, ‘APH + low PHA’). This Mcm4 dephosphorylation was reversed with caffeine, consistent with it being mediated by the checkpoint-enhanced recruitment of PP1γ to chromatin (figure 7*d,e*, ‘APH + low PHA + caffeine’).

## 4. Discussion

Using the small molecule inhibitor, PHA-767491, we demonstrate that in *Xenopus* egg extracts, Cdc7 executes its essential functions early in S phase, prior to the Cdk-dependent step in replication initiation. We show that Cdc7 is not the rate-limiting factor in driving the replication timing programme in egg extracts once Cdc7 has executed its essential function early in S phase. We also prove that PP1 rapidly reverses the Cdc7-dependent hyperphosphorylation of Mcm4, and that checkpoint-mediated enhancement of PP1 activity functionally opposes Cdc7.

### 4.1. Cdc7 is not rate-limiting for DNA replication in egg extracts

**Figure 8.**
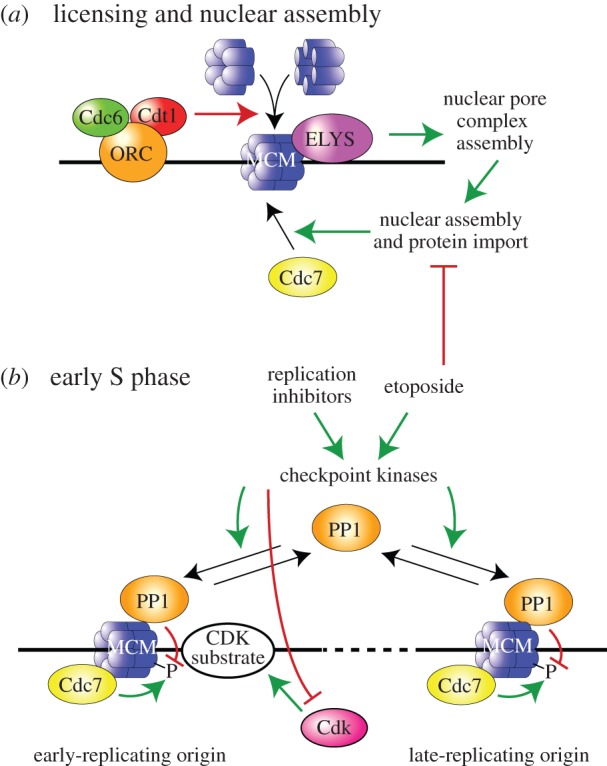
Schematic diagram of the role and regulation of Cdc7 in *Xenopus* egg extracts. (*a*) A single replication origin is shown which is licensed by loading a double hexamer of Mcm2–7 proteins (‘MCM’). This promotes ELYS binding and the recruitment of Cdc7 to Mcm2–7 at the origin. (*b*) Events occurring during S phase are shown at both an early-replicating (left) and a late-replicating origin (right). Mcm2–7 at both origins are phosphorylated by Cdc7, which in turn is reversed by PP1. Cdk substrates for initiation are recruited preferentially to the early-replicating origin. Checkpoint kinases activated in response to etoposide or other inhibitors promote PP1 chromatin association with reverse Cdc7 activity but may also independently inhibit Cdk activity.

We have used PHA-767491 to define precisely when Cdc7 acts in the sequence of events leading to replication initiation in *Xenopus* egg extracts (figure 8). On addition of sperm nuclei to egg extract, chromatin rapidly decondenses and origins become licensed with Mcm2–7 double hexamers [1,5,61]. Decondensed chromatin is then surrounded by a functional nuclear envelope, which is stimulated by licensed origins that promote the assembly of the nuclear pore precursor ELYS [53] (figure 8*a*). Cdc7 then binds and phosphorylates Mcm2–7, leading to hyperphosphorylation of Mcm4. Although nuclear envelope assembly is not strictly required for this, it stimulates both Cdc7 recruitment to chromatin and Mcm4 hyperphosphorylation. Once Cdc7-dependent phosphorylation of the Mcm2–7 complex has occurred, S phase Cdk activity can then promote initiation by phosphorylating its substrates [15,42,43].

The precise duplication of the eukaryotic genome takes place according to a reproducible replication timing programme. Origins are organized in spatially distinct clusters that fire coordinately. Although there is significant cell-to-cell variation in origin usage within clusters, the stage of S phase when specific clusters replicate is reproducibly consistent between different cells [62,63]. An attractive hypothesis is that Cdc7 preferentially associates with Mcm2–7 at early-replicating origins in early S phase, and only when these early firing origins have replicated would Cdc7 be displaced to activate later firing origins. This mechanism can explain the early initiation of pericentromeric replication origins in *Saccharomyces cerevisiae* [27]. However, our data suggest that this is not how the replication timing programme is driven in *Xenopus* egg extracts. We demonstrate that early in S phase Cdc7 executes its function on both early- and late-firing origins (figure 8*b*). This is consistent with the quantitative phosphorylation of Mcm2–7 in *Xenopus* egg extracts, which occurs even though only a fraction of these origins normally initiate in S phase [64–66]. We also prove that if MCM dephosphorylation is prevented, then the early activity of Cdc7 is sufficient to allow approximately normal progression through the replication timing programme. This is in contrast to Cdk activity, which is required throughout S phase for new initiation events to occur and for progression through the replication timing programme [49,50,56,58]. The requirement for Cdk activity to drive the replication timing programme in *Xenopus* egg extracts is consistent with the idea that Cdk substrates for initiation are rate-limiting, and are preferentially recruited to early-replicating origins (as indicated in the left-hand origin of figure 8*b*).

We suggest that the rapid phosphorylation of all replication origins by Cdc7 in *Xenopus* egg extracts is an adaptation to drive the extremely short S phases that occur in the early embryo prior to the mid-blastula transition. However, as the embryonic cell cycles become longer at the mid-blastula transition, the increasing nuclear to cytoplasmic ratio means that DDK activity also becomes limiting for replication initiation [67].

### 4.2. Cdc7 function is opposed by PP1

We demonstrate that the Cdc7-dependent phosphorylation of Mcm4 is rapidly reversed by PP1. This is consistent with a previous study which indicated that PP1 associated with Mcm2–7 complexes on chromatin assembled in *Xenopus* egg extracts [5]. Although Cdc7 phosphorylates all licensed origins early in S phase in *Xenopus* egg extracts, continued kinase activity would be required for normal S phase progression because of rapid reversal by dephosphorylation. Control of Cdc7 function by regulation of PP1 activity represents a new mechanism for regulation of DNA replication. This role for PP1 is likely conserved throughout eukaryotes, as mutation of the *S. cerevisiae* PP1 homologue Glc7 increases Mcm4 phosphorylation (S. Hiraga & A. Donaldson 2013, personal communication).

### 4.3. Cdc7 in the intra-S checkpoint response

Cdc7 has been implicated as a downstream target of the intra-S checkpoint in *Xenopus* [34]. However, subsequent studies in *Xenopus* extracts and human cells have shown that Cdc7 remains active in response to replicative stress and may be important in recovery functions [9,11,36–38]. Our demonstration that Cdc7 function is rapidly reversed by PP1 suggests that Cdc7 activity will be required for activation of dormant origins in response to replication fork inhibition [31], consistent with its proposed role in recovery from replicative stresses.

In addition, we demonstrate that etoposide, aphidicolin and double-strand DNA breaks promote the association of PP1 with chromatin in a checkpoint-dependent manner and that this leads to Mcm4 dephosphorylation. This Mcm4 dephosphorylation could be reversed by inhibiting PP1, providing further evidence that Cdc7 function is strongly restrained by PP1. Unlike the other replication inhibitors we examined, etoposide was unique in being able to strongly inhibit Mcm4 phosphorylation. This was probably owing to the fact that in addition to causing checkpoint-dependent association of PP1 with chromatin, etoposide also inhibited Cdc7 binding to chromatin in a checkpoint-independent manner. This conclusion is consistent with a previous report indicating that etoposide does not inhibit Cdc7 kinases activity [38].

Taken together, our results suggest that Cdc7 function is restrained by checkpoint kinases promoting PP1 chromatin association, thereby reversing Cdc7-mediated MCM phosphorylation (figure 8*b*). This model provides a resolution to the rather contradictory literature concerning checkpoint regulation of Cdc7 activity in metazoans [9,11,34–38]. By demonstrating a major role for PP1 in the regulation of Cdc7 activity, our work also has implications for the best use of DDK inhibitors as anti-cancer agents [39,40,68,69], in particular suggesting that modulation of PP1 activity could enhance their therapeutic efficacy.

## 5. Material and methods

### 5.1. *Xenopus* egg extract and DNA templates

Metaphase-arrested *Xenopus laevis* egg extract and demembranated *Xenopus* sperm nuclei were prepared as described [70]. Extracts were supplemented with 250 μg ml^−1^ cycloheximide, 25 mM phosphocreatine and 15 μg ml^−1^ creatine phosphokinase and incubated with 0.3 mM CaCl_2_ for 15 min to release from metaphase arrest. For DNA synthesis reactions, sperm nuclei were incubated at 6–10 ng DNA μl^−1^ or CHO nuclei at 60 ng DNA μl^−1^ in extract. When CHO nuclei were used, extracts were further supplemented with 150 μg ml^−1^ geminin [58]. DNA synthesis was assayed by measuring incorporation of [α-^32^P]dATP into acid-insoluble material followed by scintillation counting, assuming an endogenous dATP pool of 50 μM [70,71]. All incubations were carried out at 23°C.

CHO nuclei were prepared as described [58]. Briefly, asynchronous cells were treated with 50 ng ml^−1^ nocodazole (Sigma-Aldrich) for 4 h and mitotic cells shaken off, and replated for 4 h in fresh medium to obtain post-origin decision (ODP) point G1 cells. Cells were harvested, washed and permeabilized with 50 μg ml^−1^ digitonin (Sigma). The resultant nuclei were counted and added to egg extract.

### 5.2. Chromatin isolation from egg extract

Chromatin isolation for immunoblotting was carried out as described [70]. Briefly, extract was diluted with ice-cold Nuclear Isolation Buffer (NIB) (50 mM KCl, 50 mM HEPES–KOH pH 7.6, 5 mM MgCl_2_, 0.5 mM spermidine, 0.15 mM spermine, 2 mM DTT) containing phosphatase inhibitors, under-laid with NIB + 20% sucrose (w/v) and centrifuged in a swinging bucket rotor at 2100*g* for 5 min at 4°C. Following a cushion wash, chromatin was compacted by spinning at 13 000*g* for 2 min in a fixed angle rotor. The resulting pellet was resuspended in SDS loading buffer.

To isolate intact nuclei for transfer experiments, Triton X-100 was omitted from all buffers. Extracts were diluted as before, under-laid with a double cushion of NIB + 20% sucrose and NIB + 30% glycerol (v:v in NIB) and centrifuged in a swinging bucket rotor. Following a cushion wash, nuclei were resuspended in the glycerol cushion and added to the second extract at a final concentration of 10 ng DNA μl^−1^. For detection of PP1 subunits, extracts were diluted with 100 volumes ice-cold NIB and under-laid with 250 µl NIB + 30% sucrose (w/v).

### 5.3. Immunoblotting

For immunoblotting, samples were separated on 4–12% Bis–Tris gradient SDS–PAGE gels (Invitrogen). Proteins were transferred onto polyvinylidene difluoride (PVDF) membranes (GE Healthcare, RPN303F) using a wet transfer system, blocked in PBS with 0.2% Tween-20 and 5% non-fat milk. After incubation with primary and secondary antibodies, membranes were developed using enhanced chemiluminescence detection (SuperSignal West Pico Chemiluminescent; Thermo Scientific, 34087). The lower portion of each gel was typically cut and treated with Coomassie stain to visualize histones. Band intensities were quantified using GelEval (FrogDance Software). For quantification of Mcm4 hyperphosphorylation, Mcm4 bands were divided vertically into two equal halves, and the upper, hyperphosphorylated intensity was expressed as a percentage of the total Mcm4 intensity.

### 5.4. Recombinant proteins, reagents and antibodies

Geminin was produced as previously described [72]. Full length p27^kip1^ was expressed from a pGEX-p27^kip1^ plasmid (a gift of J. Walter, Harvard Medical School) and purified from Rosetta(DE3)pLysS cells (Novagen) using glutathione–sepharose. PHA-767491 [39] and I-2 were produced by the Division of Signal Transduction Therapy, University of Dundee. Roscovitine was from Calbiochem, caffeine from ICN Biochem, NU7441 from Axon Medchem and KU55933 from Tocris. Antibodies against PCNA, PP1α and PP1γ were from Santa Cruz Biotechnology. Mcm4, Cdc6 and Cdc45 antibodies were as previously described [5,46,73]. The Cdc7 antibody was raised in sheep against a bacterially expressed immunogen consisting of the C-terminal 99 amino acids of *Xenopus* Cdc7. The antisera were affinity purified prior to use (see electronic supplementary material, figure S4).

### 5.5. Fork rate analysis

For alkaline agarose gel analysis of fork rate, chromatin templates were incubated at 15 ng µl^−1^ in interphase egg extract supplemented with p27^kip1^ and [α-^32^P]dATP. Reactions were stopped with 10 volumes of stop N (20 mM Tris–HCl, pH 8, 200 mM NaCl, 5 mM EDTA, 0.5% SDS) containing 2 µg ml^−1^ RNase A. DNA was extracted with phenol : chloroform : isoamyl-alcohol (25 : 24 : 1) using Phase Lock Gel tubes (Eppendorf), ethanol-precipitated and resuspended in alkaline loading buffer (25 mM NaOH, 3 mM EDTA, 1.25% Ficoll, 0.0125% bromocresol green). Agarose gels were poured in 50 mM NaCl, 1 mM EDTA and then equilibrated in 50 mM NaOH, 1 mM EDTA for 1 h. Gels were run at 2 V cm^−1^ for 12 h and then fixed in 7% trichloroacetic acid (w/v), 1.4% (w/v) sodium pyrophosphate for 20 min. Gels were dried between sheets of 3MM paper (Whatman) and exposed to X-ray film. As DNA size standards, 25–50 ng of α-phage HindIII markers (New England Biolabs) end-labelled with [α-^32^P]dATP were run in parallel. Fork rate was estimated by determining the position of the of nascent strand peak intensity using GelEval (FrogDance Software), and then fitted by linear regression.

### 5.6. Replication pattern labelling and analysis

Somatic nuclei replication patterns were assessed as described [58]. Briefly, CHO nuclei replicating in egg extract were pulse-labelled with 25 μM Cy3-dUTP (GE Healthcare) for 2.5 min. Nuclei were isolated and fixed in 4% paraformaldehyde and spun down onto poly-l-lysine coverslips. Total DNA was stained with Hoechst 33258 and coverslips were mounted with Vectashield mounting medium and sealed. Images were acquired using a cooled camera (CoolSNAP HQ; Photometrics) on a restoration microscope (DeltaVision Spectris; Applied Precision) built around a stand (Eclipse TE200; Nikon) with a 100× 1.4 NA Plan Apo lens (Nikon). Images were taken every 0.25 μm, and 22 optical sections were recorded for every nucleus. Three-dimensional datasets were deconvoluted using the constrained iterative algorithm software (SoftWoRx; Applied Precision), and images were analysed in the Open Microscopy Environment (www.openmicroscopy.org). Timing patterns for 20 nuclei at each data point were classified as described [58] in three independent experiments.

## Supplementary Material

Supplementary Table S1: Specificity of PHA-767491

## Supplementary Material

Supplementary Figures
