# Supplementary material for: Xenopus Cdc7 executes its essential function early in S phase and is counteracted by checkpoint-regulated protein phosphatase 1
Source: Open Biol. 2014 Jan 8;4(1):130138. doi: 10.1098/rsob.130138 (PMC3909274; doi:10.1098/rsob.130138)
Supplement: Supplementary Table S1: Specificity of PHA-767491 [file rsob130138supp1.pdf]

No.	Kinase	Activity (%)	SD
1	DYRK2	3.9	0.4
2	PRK2	4.1	0.9
3	DYRK3	4.4	0.3
4	DYRK1A	5.3	0.0
5	p38d MAPK	6.3	1.7
6	GSK3b	8.4	0.3
7	CK1	9.4	1.8
8	ERK8	12.2	0.2
9	ROCK 2	13.8	1.6
10	p38g MAPK	14.3	0.6
11	AMPK	24.5	1.3
12	CDK2-Cyclin A	27.8	0.8
13	MINK1	28.9	2.2
14	GCK	30.7	1.2
15	PRAK	35.3	2.1
16	PKD1	38.1	3.4
17	IRR	39.0	1.9
18	S6K1	39.5	4.1
19	HIPK2	42.6	4.2
20	PLK1	49.2	2.4
21	MNK2	49.8	4.8
22	CHK2	50.1	2.7
23	RIPK2	53.2	5.8
24	RSK1	54.9	0.9
25	BRSK2	56.9	3.1
26	MSK1	57.0	4.1
27	TTK	57.0	3.7
28	MKK1	58.1	0.7
29	MARK3	60.0	0.4
30	MAPKAP-K2	60.3	2.0
31	CAMKKb	62.2	1.3
32	ERK2	62.9	3.9
33	ERK1	67.1	6.3
34	LKB1	70.0	2.8
35	MARK2	70.1	12.6
36	BRSK1	71.6	0.2
37	SmMLCK	71.7	6.4
38	NUAK1	74.4	1.1
39	IKKb	80.8	3.9
40	MELK	80.9	1.6
41	SGK1	82.5	2.4
42	MARK4	83.2	10.1
43	PKCa	84.8	3.6
44	PIM1	85.0	0.5
45	CK2	87.6	11.2
46	PKCz	88.0	2.7
47	RSK2	88.2	2.0
48	IKKe	88.6	1.7
49	PIM3	88.9	6.8
50	MST4	89.7	6.8
51	PKA	89.8	2.4
52	PKBa	91.6	1.3
53	CHK1	93.0	5.8
54	MST2	93.1	0.5
55	PAK2	94.6	3.0
56	PAK5	95.9	10.2
57	IRAK4	96.1	2.0
58	JNK1	97.6	6.4
59	PDK1	98.3	3.6
60	MAPKAP-K3	99.2	6.2
61	JNK3	99.3	13.0
62	Lck	99.5	2.1
63	NEK6	100.2	1.6
64	TBK1	101.3	9.3
65	PAK6	101.4	5.1
66	IR-HIS	101.5	24.4
67	VEG-FR	101.9	3.0
68	MLK3	103.3	6.3
69	Src	104.7	1.4
70	Aurora A	105.0	0.5
71	PIM2	105.0	1.3
72	HIPK3	105.2	1.7
73	p38a MAPK	105.8	4.9
74	EPH A2	105.9	0.3
75	JNK2	106.4	3.1
76	MNK1	106.5	3.0
77	IGF-1R	106.6	7.8
78	PAK4	107.3	6.2
79	MLK1	108.5	0.6
80	EF2K	108.7	0.0
81	HER4	108.8	18.0
82	PKBb	111.3	0.6
83	NEK2a	111.5	0.5
84	p38b MAPK	111.9	3.2
85	PHK	112.6	11.1
86	BTK	114.0	21.6
87	CAMK1	114.7	10.1
88	SYK	114.7	1.3
89	SRPK1	114.7	0.1
90	Aurora B	115.4	7.0
91	YES1	116.6	5.8
92	EPH-B3	118.0	11.3
93	FGF-R1	120.8	24.2
94	CSK	121.9	0.6
95	HIPK1	159.4	13.1
