# Supplementary material for: Xenopus Cdc7 executes its essential function early in S phase and is counteracted by checkpoint-regulated protein phosphatase 1
Source: Open Biol. 2014 Jan 8;4(1):130138. doi: 10.1098/rsob.130138 (PMC3909274; doi:10.1098/rsob.130138)
Supplement: Supplementary Figures [file rsob130138supp2.pdf]

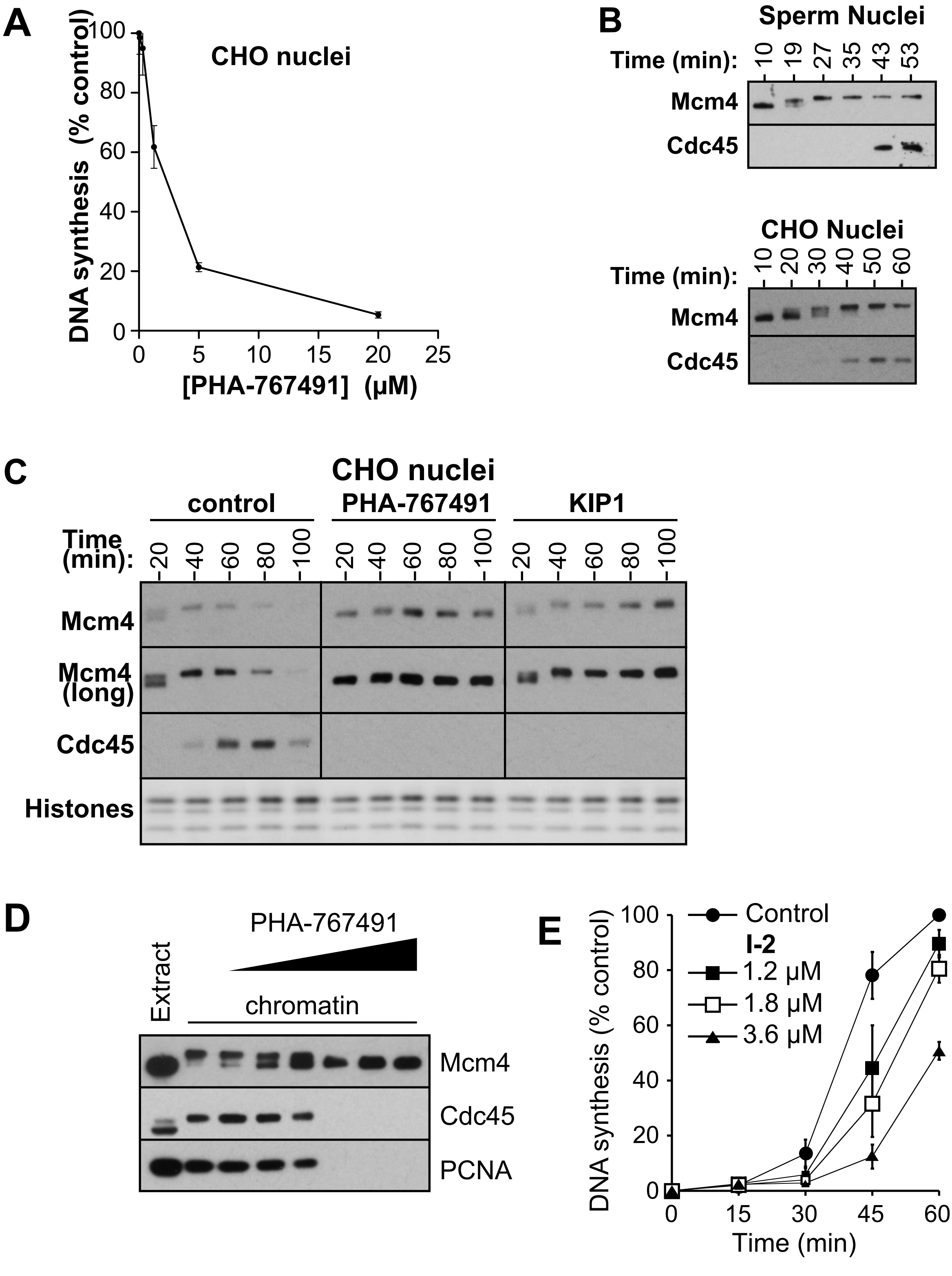

Supplementary Figure S1. Mcm4 is phosphorylated early in S phase. Demembranated *Xenopus* sperm nuclei (6 ng DNA/μl) or G1 CHO nuclei (60 ng DNA/μl were incubated in *Xenopus* egg extract. **A.** Extracts were supplemented with G1 CHO nuclei, [α-^{32}P]dATP and various concentrations of PHA-767491. After 90 min total DNA synthesis was determined. **B.** After incubation with template DNA for the indicated times, chromatin was isolated and immunoblotted for Mcm4 and Cdc45. **C.** Extracts were optionally supplemented with 50 μM PHA-767491 and p27kip1. After incubation with CHO nuclei for the indicated times, chromatin was isolated and immunoblotted for Mcm4 and Cdc45. The bottom part of the gel was stained with Coomassie to visualise histones. **D.** Extract was supplemented with different concentrations of PHA-767491 (0, 0.05, 0.2, 0.78, 3.1, 12.5 or 50 μM) chromatin was isolated after 40 min and immunoblotted for Mcm4, Cdc45 and PCNA. **E.** Sperm nuclei were incubated in extract conatining [α-^{32}P]dATP and various concentrations of I-2 (1.2, 1.8 or 3.6 μM). At the indicated times, total DNA synthesis was determined.

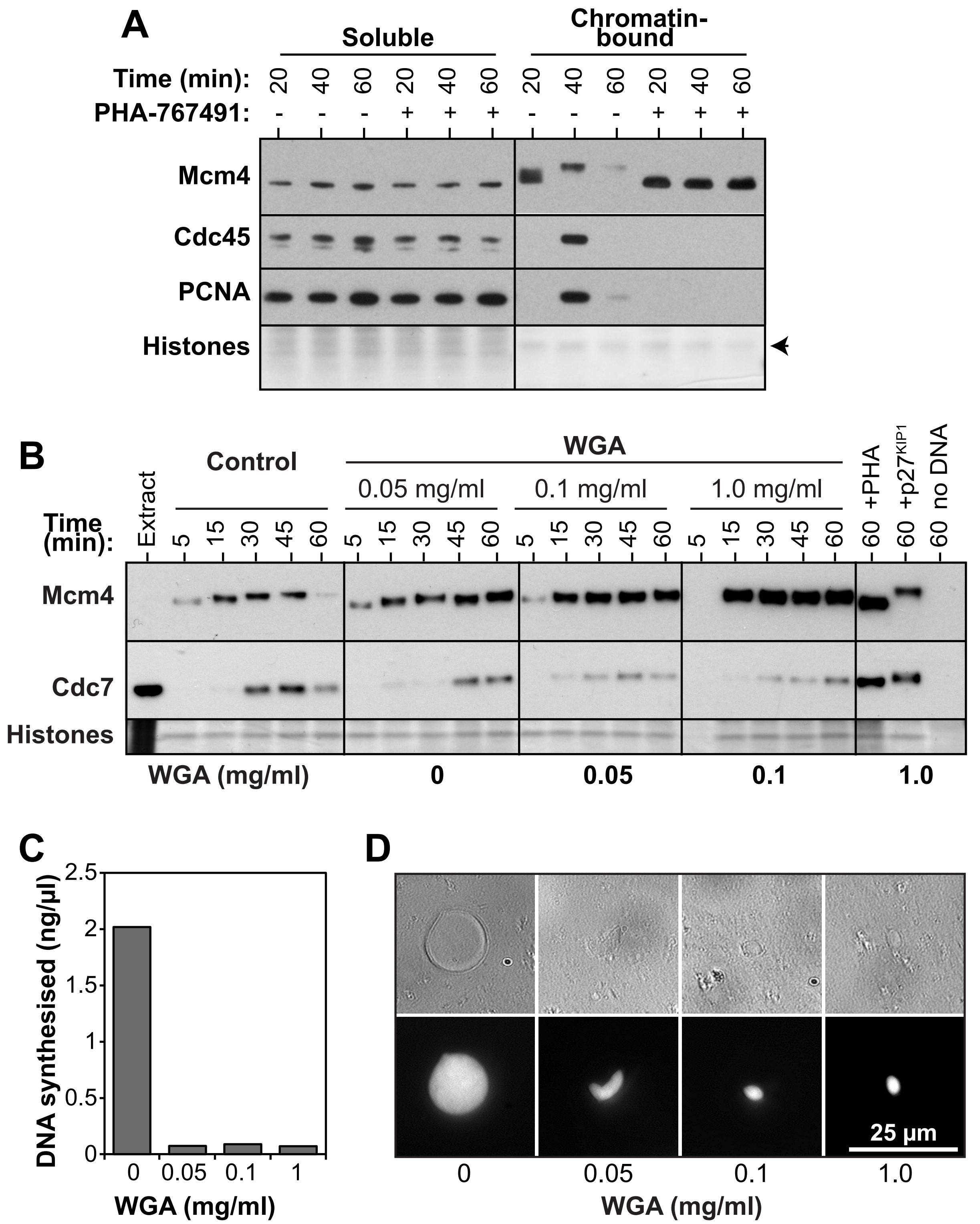

Supplementary Figure S2. Nuclear formation enhances Cdc7 activity on Mcm4.
A. Sperm nuclei were incubated at 10 ng DNA/µl in interphase extract plus or minus 50 µM PHA-767491. At the indicated times, chromatin was isolated and immunoblotted for Mcm4. 0.5% (v/v) of the supernatant was also immunoblotted. The bottom part of the gel was stained with Coomassie to visualise histones (arrowed). **B.** Sperm nuclei were incubated in interphase extract supplemented with different concentrations of WGA. At different times chromatin was isolated and immunoblotted for Mcm4 and Cdc7. The bottom part of the gel was stained with Coomassie to visualise histones. **C.** Sperm nuclei were incubated in interphase extract supplemented with [α-^{32}P]dATP and different concentrations of WGA. After 90 min total DNA synthesis was determined by scintillation counting. **D.** Sperm nuclei were incubated in interphase extract supplemented with different concentrations of WGA. At 40 min, nuclei were stained with Hoechst 33258 and visualised by phase contrast (top row) or fluorescence microscopy (bottom row).

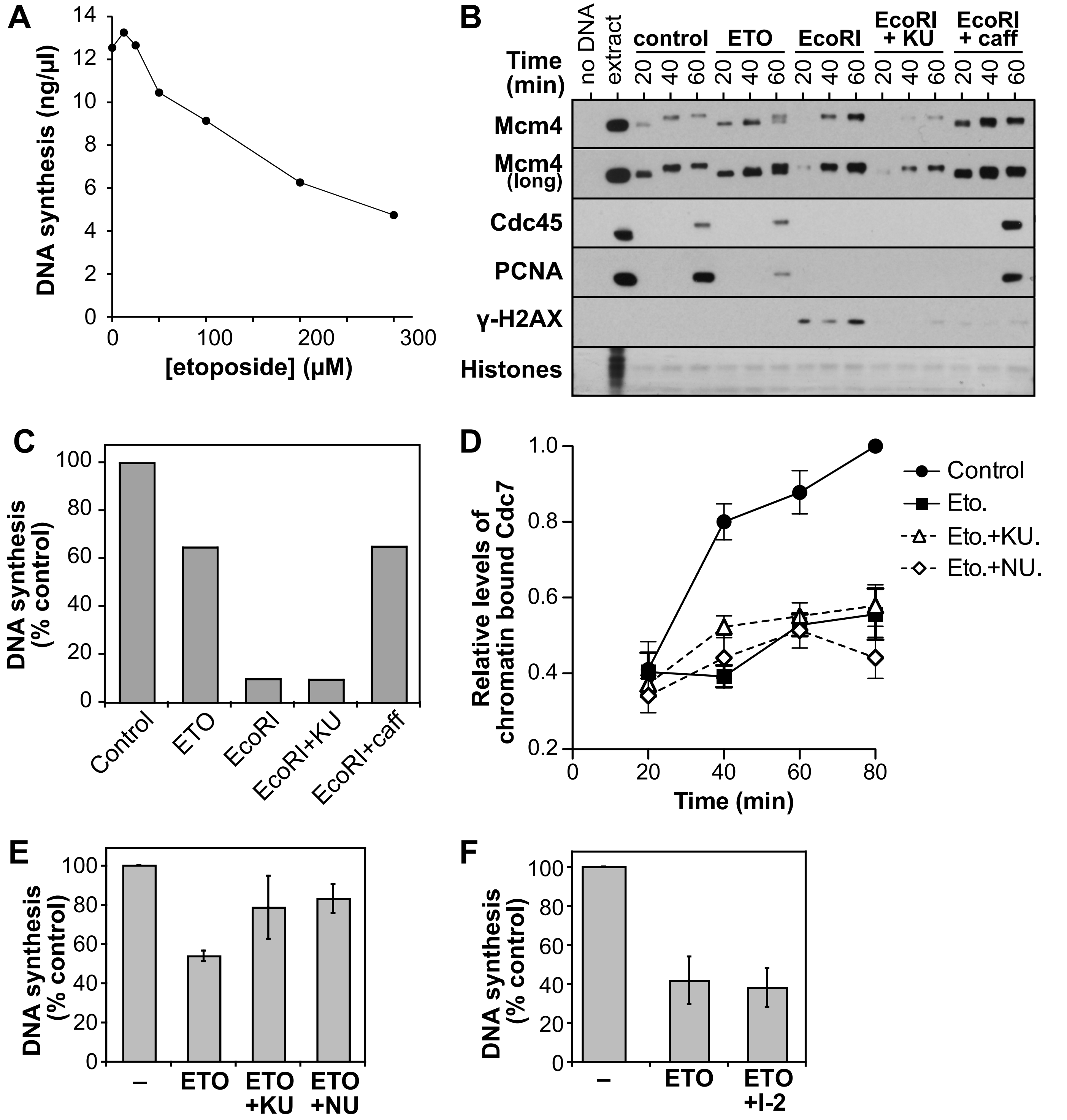

Supplementary Figure S3. Effect of etoposide and double strand breaks on Cdc7 function

A. Etoposide was titrated into extract supplemented with 12 ng/µl sperm chromatin and [α-^{32}P]dATP. After incubation for 90 min, total DNA synthesis was measured. **B, C.** Sperm nuclei were incubated in extracts treated with 300 µM etoposide, EcoRI, 10 µM KU55933 and/or 5 mM caffeine. (B) Chromatin was isolated at the indicated times and immunoblotted for Mcm4, Cdc45, PCNA and γ-H2AX. The bottom part of the gel was stained with Coomassie to visualise histones. (C) Extracts were also supplemented with [α-^{32}P]dATP and after 90 min incubation the total amount of DNA synthesised was determined. **D.** Sperm nuclei were incubated at 10 ng DNA/µl in extracts treated with 300 µM etoposide, 10 µM KU55933 and/or 10 µM NU7441. At the indicated times chromatin was isolated and immunoblotted for Cdc7. The mean and S.E.M. for the relative levels of chromatin in 3 independent experiments is shown. **E, F.** Sperm nuclei were incubated in extracts optionally treated with etoposide, 10 µM KU55933, 10 µM NU7441 or I-2. After incubation for 90 min, total DNA synthesis was measured. The mean and S.EM. of 3 independent experiments is shown.

A

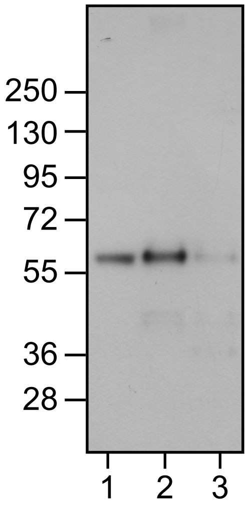

B

	Protein description	Peptide number
1.	Cell division cycle 7	41
2.	F-box-like/WD repeat-containing protein TBL1XR1-B	22
3.	Coiled-coil domain containing 138	15
4.	ATPase, H+ transporting, lysosomal 70kDa, V1 subunit A	13
5.	Drf1; DBF4 homolog B	10

Supplementary Figure S4. Characterisation of *Xenopus* Cdc7 antibody.
A. 0.5 µl of Xenopus egg extract (lane 1) and chromatin isolated from early S phase (40 minutes) ±geminin (lane 2 minus geminin, lane 3 plus geminin) were separated by SDS-PAGE and immunoblotted with affinity-purified anti-Cdc7 antibody. The migration of molecular weight markers (sizes in kDa) is shown to the left.
B. Immunoprecipitations (IP) from *Xenopus* interphase egg extract were performed using affinity-purified Cdc7 antibody or control sheep IgG (Sigma, S2763) coupled to Protein-G Dynabeads (Invitrogen). IP samples were run on a 4–12% gradient NuPAGE gel (Invitrogen). The gel was stained with SimplyBlue SafeStain (Invitrogen). Cdc7 and IgG lanes were cut into slices, samples were reduced with dithiothreitol, alkylated with iodoacetamide and in-gel digested with trypsin. The extracted peptide solutions were analysed using nano LC-MS/MS on an LTQ Orbitrap Velos (ThermoFisher, San Jose, CA). The top five hits by peptide number for proteins identified only in Cdc7 IP and not in control IgG IP are presented.
